# Singer’s Nodules: Investigating the Etiopathogenetic Markers Progressing Their Pathogenesis and Clinical Manifestations

**DOI:** 10.3390/biology10121268

**Published:** 2021-12-03

**Authors:** Mara Pilmane, Dins Sumerags, Nityanand Jain, Shivani Jain, Gunta Sumeraga

**Affiliations:** 1 Department of Morphology, Institute of Anatomy and Anthropology, Riga Stradiņš University, Dzirciema Street 16, LV-1007 Riga, Latvia; 2 Cesu Klinika Hospital, Slimnicas Street 9, LV-4101 Cesis, Latvia; dins_sumerags@yahoo.com; 3 Department of Oral and Maxillofacial Surgery, Genesis Institute of Dental Sciences & Research, Ferozepur 152002, Punjab, India; drvanijain@rediffmail.com; 4 Department of Otorhinolaryngology, Riga Stradiņš University, Dzirciema Street 16, LV-1002 Riga, Latvia; gunta.sumeraga@rsu.lv

**Keywords:** proliferation, apoptosis, growth, ischemia, inflammation, innervation, immunohistochemistry, vocal nodules

## Abstract

**Simple Summary:**

Vocal nodules, together with vocal polyps, are the most common benign vocal cord structures that are thought to be caused by extensive vocal abuse (shouting, talking loudly for prolonged periods) and are routinely treated surgically. However, surgical excision of these nodules, does not exclude the possibility of recurrence of these nodules, especially if lifestyle changes are not adapted to prevent phonetic trauma. Furthermore, the etiopathogenetic pathways governing the formation and maintenance of these nodules are not known. Herein, we investigated the etiopathogenetic markers for proliferation, apoptosis, growth, ischemia (tissue hypoxia), inflammation and innervation to elucidate the causative pathways. We found a profound and significant intensification of apoptosis in tissue epithelium, which strongly correlated with proliferative, ischemic, and inflammatory changes, highlighting the underlying complex interactions between various mechanisms on a cellular and tissue level, which occur during the morpho-pathogenesis of vocal nodules.

**Abstract:**

Vocal nodules (or Singer’s nodules) are benign vocal cord structures which are commonly encountered by clinicians. Though phonetic trauma/abuse is thought to be the main cause of the development of vocal nodules, the exact etiopathogenesis remains unknown. Hence, we compared the immunohistochemical markers for proliferation (Ki-67), apoptosis (TUNEL), growth (EGFR), ischemia (VEGF), inflammation (IL-1α and 10), and immunoreactive innervation (PGP 9.5), in vocal nodule tissue samples obtained from 10 females (17–56 years) and vocal cord tissue from seven controls. A statistically significant increase in Ki-67, TUNEL, EGFR, VEGF and IL-1α expression was noted (*p* < 0.05) between nodule tissue and control tissue in both epithelial and subepithelial layers. However, the difference was non-significant for both IL-10 and PGP 9.5 (*p* > 0.05). All markers demonstrated moderate to strong positive correlations, except for IL-10. These findings suggest increased cellular growth and proliferation in vocal nodules coupled with a persistent presence of inflammatory and ischemic environment. Furthermore, global prevalence of apoptotic cells and decreased anti-inflammatory cytokines highlight the presence of underlying complex mechanisms in the etiopathogenesis of vocal nodules, with age having a negligible impact on the marker levels. Our results could potentially further our knowledge in understanding the effects of different treatment modalities available at the cellular level.

## 1. Introduction

Neoplastic structures of the vocal cords are divided into malignant and benign lesions. Clinically, the most common benign masses/lesions of the vocal fold are polyps, nodules, Reinke’s oedema, and cysts, and are thought to be due to phonetic trauma [1]. The presence of vocal noduli in the larynx leads to increased friction of the vocal cords against each other, thereby raising the circumscribed hyperplastic changes and/or hyperkeratinisation of the epithelial layer, coupled with secondary hyaline-degeneration in the lamina propria of the vocal cords. Vocal noduli, together with vocal polyps, are caused by extensive vocal abuse (shouting, talking loudly for prolonged periods) and are routinely treated surgically [2]. However, surgical excision of these nodules does not exclude the possibility of their recurrence, especially if lifestyle changes are not adapted to prevent phonetic trauma.

Previously, multiple studies have been reported that describe the routine histomorphology of the vocal noduli, however, most of them lack a specific description or exploration of the various possible morpho-pathogenetic events that might be involved in the development and maintenance of vocal noduli, such as proliferative, programmed cell death, growth, ischemic, inflammatory, and neuropeptide-containing innervation pathways and proteins. Under physiological conditions, the tissue of the vocal box is constantly undergoing renewal and regenerative processes due to the permanent stress it endures while producing voice. Moreover, the rate and intensity of these processes in the tissue depend upon a person’s functional intensity of their vocal apparatus, with more frequent and abusive use accelerating these processes.

The main proliferation marker used for histomorphological analysis is the human Ki-67, an antigen encoding two related protein isoforms. The Ki-67 protein is present in all active stages of a cell cycle (G1, S, G2, M), but is absent in the non-active stages (G0) [3,4]. Thus, it is an extremely reliable and useful marker for determining the growth fraction of a cell population and is often used as an indicator for proliferation intensity, for example, to estimate a tumour’s proliferation index [5]. Consequently, an even more prognostic value for the marker is evident in various neoplasms of the soft tissue, breast, lung, prostate, cervix, and central nervous system [6,7,8,9]. Though the marker is extensively employed in oncology, its expression in the healthy vocal cord tissue and benign structures has been studied in a limited context.

Several voice disorders have been shown to be associated with the process of apoptosis or programmed cell death. The most common of such epithelium-associated apoptotic processes are described in the context of laryngeal squamous cell carcinoma, leucoplakia, keratosis, epithelial hyperplasia, human papilloma virus (HPV) papilloma and few other benign lesions. Hence, this leads us to speculate that apoptotic cell death may at least partly contribute to the pathogenesis of diseases affecting the vocal fold epithelium [10]. Additionally, apoptosis has been described in the laryngeal epithelium in response to the physical stressors of abduction, adduction, and biochemical vibrations. The authors reported the persistence of a moderate number of apoptotic cells, whilst a slower cellular turnover was described, for instance, during the immobilization of vocal folds [10]. Interestingly, by evaluation of the apoptotic index in the vocal fold polyps, a higher apoptotic index was established in another study. The authors further reported simultaneous intensification of mitotic divisions, inflammation, and exocytosis when compared with the normal epithelium of the vocal folds [11]. However, the calculations of the apoptotic index and, in general, the apoptotic process in the case of vocal cord nodules, have not been established until now.

The development of vocal cord nodules is usually stimulated by the intensification of the local blood supply and associated local stimulating factors. To these factors belongs the vascular endothelial growth factor (VEGF), an angiogenic factor described as one of the most important and critical growth factors required for neoangiogenesis in the vascular endothelial cells. VEGF is produced by many cell types including tumour cells, macrophages, platelets, keratinocytes, renal mesangial cells, and is commonly seen upregulated in many tumours where it strongly supports tumour angiogenesis and survival. Apart from playing a role in the vascular system, VEGF is also active during multiple normal physiological and homeostatic processes such as bone formation (osteogenesis), haematopoiesis, wound healing, and development [12]. In case of tissue trauma or inflammation, different cell types can release VEGF, with tissue hypoxia or ischemic changes being one of its most potent inducers. In other words, hypoxia can regulate the cellular responses and, in turn, VEGF expression [13]. However, the role of such hypoxic conditions in vocal nodules has not been described.

Interleukin-1 (IL-1) is a member of a family of 11 interleukins, whereby IL-1 has been described to be the most potent and persistent interleukin in the settings of acute and chronic inflammations. In human diseases, IL-1 mediates the inflammatory processes by binding to the ligand-binding chain of its dedicated receptor (IL-1RI), leading to the activation of a co-receptor chain (IL-1RAcP) and the formation of a complex (IL-1RI–IL-1–coreceptor). The release of IL-1 is considered as a multifactorial process [14], under the control of multiple local and systemic factors. The release of IL-1 and prostaglandin E2 (PGE-2) in response to vocal cord injuries has been described in a rabbit model. Injuries of the vocal fold epithelium led to the release of both mediators. The concentration of both inflammatory mediators was reported by the authors at six different time points after laryngeal vocal fold injury using enzyme-linked immunosorbent assay (ELISA). The released mediators behaved in a predictable and comparable fashion to other controlled injury tissues, thereby suggesting that the surface secretions of these mediators from the vocal folds can serve as potential indicators of wound healing processes [15].

This illustrated that IL-1 as a histological marker, can be used in the same way as it is used for evaluation of other tissue specimens from the human body. Furthermore, IL-1 mediated inflammation can lead to scar formation, collagen deposition and, in most probabilities, also to the formation of vocal cord noduli. Vocal fold nodules contain a fibrin matrix organized with other matrix proteins, including collagen, and demonstrate prominent tissue fibrosis [16]. So, the composition of collagen tissue could deliver traces of the origin of the vocal cord nodule and answer the question if IL-1 is/was histologically present in the vocal noduli to mediate the pathogenesis of formation of vocal cord noduli.

One of the strongest anti-inflammatory cytokines, IL-10, was found to be increased significantly during wound-healing, stimulating acupuncture after phono-traumatic vocal fold lesions [17], with prominent expression observed also in the cases of acute vocal cord injury [18]. Moreover, IL-10 might be of particular interest in determining the prognosis of vocal cord leucoplakia as it is related to malignancy [19], but its expression in healthy vocal cords and tissue affected by vocal noduli is still not known. Another rather more interesting issue is the innervation of the vocal cord tissue that can change in different circumstances such as inflammation under the influence of various environmental factors. The rich presence of general neuroendocrine markers in the human nasal mucosa, soft palate, and the larynx [20] makes evaluating the neuropeptide-containing innervation in vocal noduli quite intriguing. Furthermore, other reports have described such nerve endings related research only in animals [21,22] and, hence, nothing is known about the changes of immunoreactive innervation in benign human vocal cord structures.

The epidermal growth factor receptor (EGFR) is a transmembrane growth factor receptor protein, which directs the behaviour of epithelial cells and tumour cells of epithelial origin. Several types of cancers demonstrate overexpression of EGFR due to the presence of a higher amplitude and frequency of its signalling. This allows the cells to proliferate much faster, aggressively, and more invasively. Therefore, an increased level of EGFR, especially in tumour cells, is connected to a poor prognosis and reduced life expectancy [23]. Since the region of the vocal box is constantly under stress for sound production, the EGFR and related tissue growth was specifically evaluated in a study associated with neoplasms during squamous cells carcinogenesis. In the study, the authors obtained specimens that ranged from normal to hyperplastic lesions with no atypia, low-grade atypia, and high-grade atypia. The authors reported that low and high-grade dysplasia had higher EGFR immunopositive cells compared to normal epithelium and simple hyperplasia. Furthermore, they established a direct positive trend between EGFR expression and the grade of the tumour [24].

Another study revealed that the vocal cord polyps demonstrate hyperplasia in 50.75% of the investigated cases, as against 82.14% in cases of vocal cord nodules [25]. At the same time, the study reported that 31.34% of polyps show atrophic changes, whilst 12.5% of vocal nodules showed such changes [25]. Generally, since the EGFR is referred to as the most important diagnostic marker in dysplastic changes, it becomes essential to also detect EGFR expression in normal vocal cord nodules. On the basis of the above presented points, the aim of our present work was to investigate the etiopathogenetic markers for proliferation, programmed cell death (apoptosis), growth, ischemia, inflammation, and immunoreactive innervation in the vocal cord noduli and normal vocal cord tissue (controls), and elucidate their possible involvement in the morpho-pathogenesis of this frequently reported clinically benign disease.

## 2. Materials and Methods

### 2.1. Patient Material Collection

Tissue samples from vocal nodules were obtained from 10 female patients who underwent surgical treatment. The females were aged between 17 and 56 years. Patient inclusion criteria were: (i) Diagnosis of chronic hyperplastic laryngitis with presence of vocal nodule; (ii) Absence of any other larynx pathology and (iii) No past/current use of medications or other treatment modalities, including previous surgeries in laryngeal region. Patient exclusion criteria included: (i) Refusal to participate in the study; (ii) Known respiratory oncological diseases, especially in larynx and (iii) Newly diagnosed or pre-oncological lesion of the respiratory tract.

The 10 female patients who were enrolled in the present study reported hoarseness in the voice for more than two weeks and were involved in voice-intensive jobs such as teaching, professional singing, etc. The patients had no significant medical history prior to this and were completely healthy. They did not visit the doctor and waited for the hoarseness to disappear on its own. However, when the symptoms did not improve after two weeks, they paid their first-ever visit to the ENT clinic, where the diagnosis of vocal noduli was provided. None of the patients were smokers, had gastroesophageal reflux disease (GERD), or any other causative aetiology for vocal noduli except voice abuse. All patients were treated in the outpatient department of the clinic and did not underwent any voice therapy. Additionally, control tissue was obtained from vocal cords of seven cadavers (aged 40–70 years) stored in the tissue collection at the Department of Morphology, Riga Stradins University (RSU).

### 2.2. Ethical Approval

The present study was conducted at the Department of Otorhinolaryngology and Department of Morphology of Riga Stradins University (RSU) between 2019 and 2020. The study protocol was approved by the local ethics committee of the Riga Stradins University (dated 31.10.2019 vide no.6–2/9/25). All the patients or parents of patients gave their informed consent to participate in the study. The nature of the study had been fully explained to the patients and parents of the patients.

### 2.3. Routine Staining and Immunohistochemistry

All collected tissue samples were fixed in a mixture of 2% formaldehyde and 0.2% picric acid in 0.1 M phosphate buffer (pH 7.2) and rinsed in Tyrode buffer (content: NaCl, KCl, CaCl_2__2H_2_O, MgCl_2__6H_2_O, NaHCO_3_, NaH_2_PO_4__H_2_O, glucose) containing 10% saccharose for 12 h, before being embedded into the paraffin wax. Thin sections (5 µm) of the paraffin blocks were cut, which were then stained with haematoxylin and eosin (H&E) for routine morphological evaluation. The Biotin–Streptavidin biochemical method was used for immunohistochemistry (IMH) to detect various immunohistochemical markers (Table 1).

Negative and positive controls were included for all antibodies to avoid background staining and non-specific binding of secondary antibodies. For negative controls, primary antibodies were not added (water controls), thereby obtaining slides with no staining. Tissues known to contain molecular factors, either from the manufacturer’s recommendations or from previous studies conducted at the Department of Morphology, RSU, were used as positive controls.

### 2.4. TUNEL Reaction

The detection of apoptotic cells was carried out using terminal deoxynucleotidyl transferase dUTP nick end labelling (TUNEL). It was performed using a standard in situ cell death detection kit, POD cat. no 11684817910, manufactured by Roche Diagnostics, in a working dilution of 1:10 [26].

### 2.5. Immunohistochemistry Semi-Quantification and Visualization

The slides were analysed by light microscopy by two independent morphologists using the previously described semi-quantitative method [27]. The results were evaluated by grading the appearance of positively stained cells in the visual field (Table 2). For a visual illustration (microphotographs), Leica DC 300F digital camera and image processing and analysis software, Image-Pro Plus (Media Cybernetics, Inc., Rockville, MD, USA) were used.

### 2.6. Statistical Analysis

Data processing was performed using the SPSS software v22.0 (IBM Company, Chicago, IL, USA). Spearman’s rank correlation coefficient was used to determine correlations between factors, where r = 0–0.2 was assumed as a very weak correlation, r = 0.2–0.4 a weak correlation, r = 0.4–0.6 a moderate correlation, r = 0.6–0.8 a strong correlation, and r = 0.8–1.0 a very strong correlation. To analyse the control group versus patient data, the Mann–Whitney *U* test was used. The level of significance for tests was chosen as 5% (*p*-value < 0.05).

## 3. Results

### 3.1. General Appearance of the Vocal Noduli Tissue

**Figure 1:**
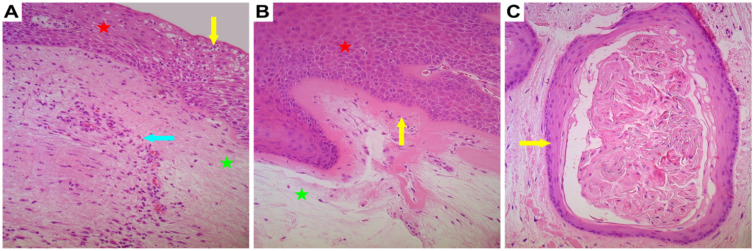
Microphotographs of routinely stained vocal noduli tissue using haematoxylin and eosin (H&E) staining. Red star indicates epithelial layer while green star indicates subepithelial layers. (**A**) Vacuolization of the epithelial cells is seen (yellow arrow) along with subepithelial infiltration of inflammatory cells (blue arrow); (**B**) Basal cell hyperplasia and thickened basal membrane in vocal noduli can be visualized (yellow arrow) and (**C**) Cyst with partially dendritic material of vocal noduli was seen in two patients (yellow arrow). Original magnification, 200×.

All vocal nodules were covered by stratified squamous epithelium and showed notable basal cell hyperplasia and vacuolized epitheliocytes (Figure 1A). Additionally, the basal membrane showed thickening, although the sub-epithelium demonstrated non-homogenous distribution of inflammatory cells, oedema, fibrotic tissue, and neoangiogenesis (Figure 1B). In tissue material from two patients, large cysts were visualized in the sub-epithelial layers (Figure 1C).

### 3.2. Vocal Noduli Showed Significantly Increased Epithelial Proliferation

**Figure 2:**
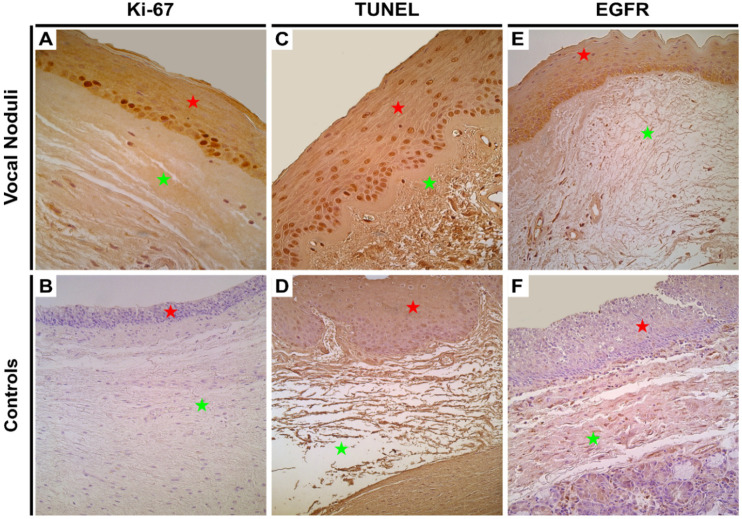
Immunohistochemical assessment of epithelial proliferation, apoptosis, and growth in the vocal noduli and control tissue. Red star indicates epithelial layer while green star indicates subepithelial layers. (**A**) Moderate number of Ki-67 positive basal epitheliocytes in the epithelial layer of a patient with vocal noduli. Original magnification, 400×; (**B**) No Ki-67 immunopositive structures are visualized in the control tissue. Original magnification, 200×; (**C**) Numerous apoptotic epitheliocytes are seen in the tissue from patient with vocal noduli using TUNEL reaction. Original magnification, 400×; (**D**) There is absence of apoptotic cells in the epithelium of vocal cord tissue obtained from controls using TUNEL reaction. Original magnification, 200×; (**E**) Numerous EGFR positive epitheliocytes along with connective tissue cells are visualized in the vocal noduli tissue. Original magnification, 250× and (**F**) Moderate number of EFGR positive connective tissue cells but no positive epitheliocytes can be seen in the control tissue. Original magnification, 250×.

Ki-67, the marker for proliferation, showed the presence of a moderate number of positive proliferating basal epitheliocytes (++) in the vocal noduli tissue, whilst the controls did not express the marker at all (0–0/+) (Figure 2A,B). Additionally, the difference between vocal noduli tissue and controls was found to be statistically significant (*p* = 0.0003; Table 3).

### 3.3. Vocal Noduli Showed Significantly Increased Epithelial Apoptosis

TUNEL reaction was used to visualize the presence of apoptosis in the tissue samples. In the vocal noduli tissue, numerous cells were observed undergoing programmed cell death (+++) compared with only a few to moderate (+/++) number of cells in the epitheliocytes from the control tissue (Figure 2C,D). The difference again, was found to be statistically significant (*p* = 0.0006; Table 3).

### 3.4. Vocal Noduli Showed Significantly Increased Epithelial Cellular Growth

EGFR was used to understand the differences in the cellular growth (hyperplastic changes). As expected, EGFR staining revealed numerous positive (+++) epithelial cells in vocal noduli tissue, whilst in controls, the staining showed positivity only in moderate number (++) of cells (Figure 2E,F). The difference was found to be statistically significant (*p* = 0.005; Table 3).

### 3.5. Vocal Noduli Tissue Showed Significant Ischaemic Compensatory Changes

**Figure 3:**
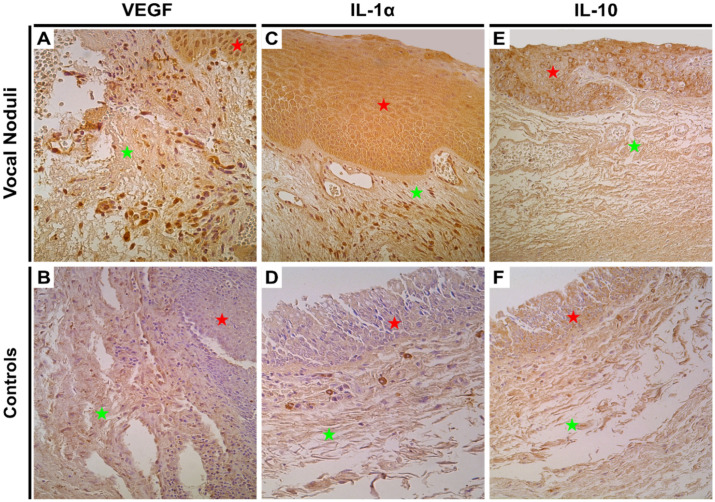
Immunohistochemical assessment of tissue ischaemia, and inflammatory environment in the vocal noduli and control tissue. Red star indicates epithelial layer while green star indicates subepithelial layers. (**A**) Numerous positive VEGF positive cells were observed in both epithelium and blood vessels of vocal noduli tissue. Original magnification, 400×; (**B**) Few positive endotheliocytes in the blood vessel of vocal cords show VEGF positivity in the control tissue. Original magnification, 250×; (**C**) Numerous IL-1α immunopositive epitheliocytes and connective tissue cells in vocal noduli tissue. Original magnification, 250×; (**D**) Few IL-1α positive macrophages are seen in the control tissue. Original magnification, 250×; (**E**) Moderate IL-10 immunopositive epitheliocytes are seen in the vocal noduli tissue. Original magnification, 200× and (**F**) Weakly stained IL-10 positive epitheliocytes and connective tissue cells (mainly macrophages) in the vocal cords from controls. Original magnification, 200×.

VEGF, an indirect marker of tissue hypoxia, was used to look for tissue stress and the associated compensatory changes (neoangiogenesis). Numerous VEGF immunoreactive cells were observed (+++) in vocal noduli tissue, but an absence of immunoreactivity was noted in the control tissue (0–0/+). Such changes were visible in both epithelium and blood vessels (Figure 3A,B). Statistically, the difference in both endothelium and epithelium was found to be significant (*p* = 0.0003; Table 3).

### 3.6. Vocal Noduli Showed Presence of Pro-Inflammatory Environment

IL-1α, a pro-inflammatory cytokine and IL-10, an anti-inflammatory cytokine, were checked to assess the tilt in the balance of inflammatory environment. We observed numerous IL-1 positive (+++) epithelial and connective tissue cells in the vocal noduli tissue whilst the controls demonstrated only few positive cells (0/+). In fact, in controls these few positive cells were tissue macrophages seen in the connective tissue (Figure 3C,D). The difference between control and vocal noduli tissue was statistically significant (*p* = 0.0003; Table 3). For IL-10, marked variation amongst vocal noduli samples was noted with an average of moderate number of positive cells (Figure 3E,F). In the control tissue, the expression of IL-10 was rather more homogenous and consistent (+/++). There was no statistically significant difference noted in expression of IL-10 (*p* = 0.050; Table 3). These findings indicate a shift in the balance towards a pro-inflammatory environment.

### 3.7. No Significant Difference in Neural Innervation Was Found in Vocal Noduli

**Figure 4:**
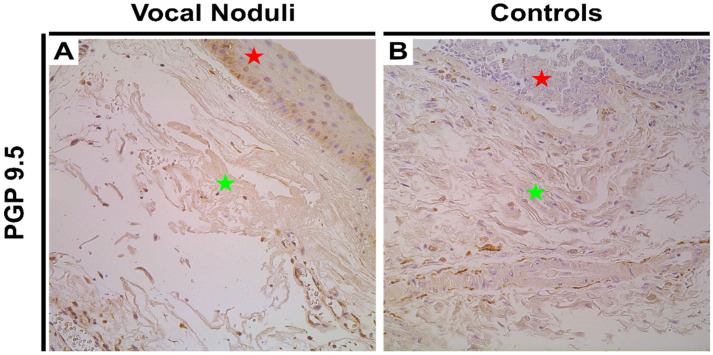
Immunohistochemical assessment of immunoreactive innervation in the vocal noduli and control tissue. Red star indicates epithelial layer while green star indicates subepithelial layers. (**A**) Few PGP 9.5 immunopositive neuroendocrine cells in the epithelium and fine nerve fibres in connective tissue can be visualized in the vocal noduli tissue. Original magnification, 200× and (**B**) Few PGP 9.5 immunopositive nerves in the subepithelium, mainly seen amongst the smooth muscle bundles of the blood vessel in the control vocal cord tissue. Original magnification, 200×.

PGP 9.5, a marker for neurons and neuroendocrine cells in non-neoplastic tissue, showed variable expression in vocal noduli tissue ranging from complete absence to presence of moderate immunoreactive nerve fibres (0 to ++). In the controls, few immunopositive neuroendocrine cells in the epithelium (+) and few nerve fibres in the subepithelium (+) were noted (Figure 4A,B). The difference was found to be statistically insignificant (*p* = 0.091; Table 3).

### 3.8. Correlation Analysis between Different Immunohistochemical Markers

A significantly strong correlation was observed between expression of Ki-67 and presence of apoptosis (ρ = 0.888, *p* < 0.001), VEGF (ρ = 0.884, *p* < 0.001), EGFR (ρ = 0.839, *p* < 0.001), and IL-1 in both epithelial (ρ = 0.834, *p* < 0.001) and connective tissue (ρ = 0.899, *p* < 0.001) in the vocal noduli tissue (Table 4). Furthermore, a significantly strong positive correlation was seen between apoptosis marker and VEGF (ρ = 0.802, *p* < 0.001), and IL-1 in both epithelial (ρ = 0.786, *p* < 0.001) and connective tissue (ρ = 0.820, *p* < 0.001) in the vocal noduli tissue.

TUNEL positive structures also strongly correlated with EGFR (ρ = 0.753, *p* < 0.001), whilst EGFR expression correlated strongly with the expression of VEGF (ρ = 0.750, *p* < 0.001). There was a strong positive correlation between epithelial IL-1 and connective tissue IL-1 (ρ = 0.868, *p* < 0.001) expression. VEGF and IL-1-containing epitheliocytes (ρ = 0.889, *p* < 0.001) and IL-1 positive connective tissue cells (ρ = 0.745, *p* < 0.001) also showed significantly strong positive correlation.

Finally, we observed a moderate positive correlation between cellular expression of Ki-67 and PGP 9.5 nerves (ρ = 0.504, *p* = 0.032) and expression of VEGF and PGP 9.5 (ρ = 0.614, *p* = 0.006). On the other hand, EGFR moderately correlated with IL-1 in epithelial (ρ = 0.625, *p* = 0.005) and IL-1 in connective tissue (ρ = 0.611, *p* = 0.007). In our present study, we also observed a weak yet significant correlation between expression of epithelial IL-1 and PGP 9.5 nerves (ρ = 0.469, *p* = 0.049).

## 4. Discussion

In the present study, we observed that vocal cord noduli demonstrated a significant and prominent increase in epithelial EGFR, VEGF, IL-1 expression, along with an increase in the global prevalence of epithelial apoptotic cells, a phenomenon not observed in the tissue samples from healthy human vocal cords. Furthermore, in the abundance of inflammatory cells in the subepithelium, notable expression of IL-1 and VEGF, and the elucidation of strong correlations between them suggest a possible connection between persisting inflammatory changes in the vocal noduli tissue, as evidenced by localized tissue hypoxia/ischemia. Indeed, not only does the repeated stress and trauma, resulting from vocal cord overuse lead to a formation of a hypoxic environment, but also the release of pro-inflammatory cytokines can initiate/maintain the development of such benign vocal cord structures. This is supported by evidence from other researchers, who reported that the hypoxia inducible factor-1α (HIF-1α) and VEGF are notably elevated in the vocal polyp tissue. The authors had hypothesised that this increased level of HIF-1α is a result of over-vibration of the vocal cord, which induced hypoxia and was followed by increased expression of VEGF [13].

The significance of closely related VEGF-EGFR intercorrelations and their activities were also described by Guo et al., who pointed out that the inhibition of this interaction may play a crucial role in some tumour proliferation signal pathway blockage [28], as the VEGFR-EGFR relation directly influences the inhibition of cell invasion and migration during the experimental conditions of wound healing. Additionally, IL-1 is the most prominent interleukin in acute and chronic inflammation settings which is released in case of endothelial injury/stimulation. Apart from vascular injury, stimulation of IL-1 by the expression of VEGF in our patients is another factor to consider in the etiopathology of vocal cord noduli [29]. We hypothesize that IL-1 mediated inflammation can aggravate the already described nodular epithelial hyperplasia, basement membrane thickening, oedema, and fibrotic changes [16]. These processes can accelerate scar formation, extra collagen deposition and maintenance/stimulation of vocal cord nodule formation.

The routine morphological findings of non-specific changes in the epithelial tissue (basal cell hyperplasia, vacuolization), together with the dominance of the above-mentioned upregulated expression of EGFR, IL-1 and Ki-67, and global apoptosis in the vocal nodule’s epithelium, suggest epithelium cells to be more intensively affected in the case of vocal abuse. The close correlations found between these factors highlight their possible role into the modulation of each other’s functions. A similar finding was observed in the animal vocal cord undergoing regeneration of its epithelium during the acute phase of injury [30]. A time-dependent secretion of the proliferation marker, Ki-67, growth factors EGF and TGFβ1, as well as activation of the EGFR was observed in the animal vocal cord [30]. The authors proposed the epitheliocytes activity in the wound healing process was guided by autocrine and paracrine signalling pathway during wound healing. We speculate on the similar lines, that in the vocal noduli of our patients, the epithelium significantly expresses different tissue factors in response to the regular phono-trauma and persistent inflammatory environment.

The expression of the human Ki-67 is closely associated with cell proliferation. Studies on vocal polyps have shown that an increase in and correlation of Ki-67, EGFR, and some other factors immunohistochemically in epithelium may change the vocal cord region. Subsequently, high-grade dysplasia also shows statistically higher expression and immunoreactivity of Ki-67, whereas low-grade dysplasia demonstrates the opposite results [24,31]. This indicated a positive expression grade of Ki-67 in vocal cords leucoplakia with epithelial dysplasia as a valuable marker for the evaluation, diagnosis, and prognosis of precancerous lesions in the vocal cords. We report here an increase in Ki-67 positive cells and thus, stimulation of epithelial proliferation in benign vocal cord noduli that occurs probably in response to specific stimulation of other intra-epithelial factors such as EGFR, VEGF, and IL-1. Intensive apoptosis was also seen in our patient’s vocal noduli epithelium. Taking into the account the close correlations between the TUNEL, VEGF, IL-1, and EGFR positive cells, we suppose the direct complex stimulation of ischemia, pro-inflammatory cytokines, and cellular proliferation inducers on the acceleration of programmed cell death in the vocal cord noduli.

Apoptosis is considered an organized/programmed pathway for cellular degradation for several reasons. Some evidence of apoptosis in the vocal fold epithelium has been reported in the past, described especially in response to biomechanical trauma which caused a rise in apoptotic rate ultra-structurally in the vocal fold epithelium after experimental vibration of rabbit vocal folds [10]. However, some contrary data revealing no significant impact of the biomechanical strength on the vocal cord cellular apoptosis has been reported as well. Human vocal cord vibrations were found to not alter the apoptosis rate after the influence of intense vibration and strain [32], which leaves the question about the origin of apoptosis in the vocal cords partially open. Most likely, in our opinion, the programmed cell death mechanisms are more complex and under multiple regulatory processes. So, the pathogenesis of vocal pathology may be associated with cell signalling miscommunication that involves apoptosis and/or irregularities in apoptosis may eventually evolve into epithelial hyperplasia and/or benign nodular formations [33,34]. The abnormal apoptosis might also be connected to the changes in the epithelial tight junctions that leads to changes in the phenotype of the epithelial cells following their irregular removal [35].

Variability in the expression of the neuropeptide-containing innervation represented by PGP 9.5 and of anti-inflammatory cytokine IL-10 in vocal noduli tissue did not differ from their expression in healthy vocal cord tissue significantly, thereby allowing us to exclude their role from the pathogenesis of vocal noduli formation. However, the variations and even absence of IL-10 expression in the patient’s vocal noduli does not necessarily remove the question about the suppression of the main anti-inflammatory cytokine in the vocal noduli tissue and probably needs a more personalized approach to the analysis of the palisade of pro-, regulatory-, and anti-inflammatory cytokines to explain their varied role and function in the persistent inflammation in these benign structures of the vocal cords.

The structural changes in the vocal noduli tissue of the patients in the age range from 17 to 56 years are quite similar thereby implying no significant effects of ageing related changes. The tissues gave similar response to different environmental irritants, and we speculate that this morphological phenomenon takes place also in the vocal cords. Additionally, in the literature only the vocal cord atrophy is described in geriatric population which shows a different morpho-pathogenesis when compared with the benign vocal box structures [36,37].

With respect to the involvement of proliferation markers, apoptosis, specific cytokines, and neo-angiogenetic factors in the morpho-pathogenesis of vocal noduli described by us, there are some limitations of our work which should be mentioned for future research. An important issue might be the tissue factor concentration revealed by ELISA and the evaluation of some gene/gene or protein/protein impact in the development/maintenance of vocal noduli. Additionally, a comparison of childhood (before the puberty age) and very old age vocal benign structures should be done to clarify the possible impact of aging on these structures. Nonetheless, this last limitation is rather very strictly limited due to the ethical considerations and due the difficulty in accessing such tissue material. Finally, the relatively small number of participants from the same gender included in the present study may limit our capacity to generalize our findings clinically. However, it is important to take into notice that the present study aimed to elucidate the patho-morphogenesis of vocal noduli at a cellular and morphological level. Future studies with a bigger study group and including both genders should be undertaken to fully understand the interplay of various underlying factors in the aetiology of vocal noduli formation and maintenance.

## 5. Conclusions

The significantly upregulated expression of Ki-67 and EGFR in the epithelial tissue suggests the intensification of cellular growth and proliferation, leading to hyperplastic changes in the tissue structure. Such changes occur in the presence of a persistent pro-inflammatory environment (significantly increased expression of IL-1α) coupled with associated ischemic changes which triggers a compensatory reaction from epithelium and endothelium alike. This causes the upregulation of angiogenesis (significantly increased VEGF expression) in the vocal noduli tissue.

Intensification of apoptosis and its strong correlation with proliferation, ischemia, and inflammatory factors highlight the underlying complex interactions between various mechanisms on a cellular and tissue level, which occur during the morpho-pathogenesis of vocal nodules and most likely are responsible for the maintenance of their formation. A similar expression of anti-inflammatory cytokine IL-10 and neuropeptide-containing innervation marker PGP 9.5 in the healthy and vocal noduli tissue eliminates the “acute” participation of these markers in the morpho-pathogenesis of the vocal nodules. Finally, the morphology of vocal nodules does not seem to depend on the age of the patient, at least in the 17–56 years age group.

## Figures and Tables

**Table 1:** Description and characteristics of the primary antibodies.

Primary Antibody *	Antibody Characteristics **	Clone	Dilution	Catalogue No.	Manufacturer
Ki-67	Monoclonal rabbit AB against human AG	SP6	1:100	1325506A	Cell Marque (USA)
IL-1α	Polyclonal rabbit AB against human AG	-	1:100	orb308737	Biorbyt (UK)
IL-10	Polyclonal rabbit AB against human AG	-	1:100	250713	Abbiotec (USA)
VEGF	Polyclonal rabbit AB against human AG	-	1:100	orb191500	Biorbyt (UK)
PGP 9.5	Polyclonal rabbit AB against human AG	-	1:100	439273A	Invitrogen (USA)
EGFR	Monoclonal mouse AB against human AG	0.N.26	1:100	sc-71034	Santa Cruz (USA)

* Abbreviations: Ki-67—marker of proliferation KI67; IL-1α—interleukin-1α; IL-10—interleukin-10; VEGF—vascular endothelial growth factor; PGP 9.5—protein gene product 9.5 and EGFR—epidermal growth factor receptor. ** Abbreviations: AB, antibody; AG, antigen.

**Table 2:** Description of the semi-quantitative method used in the present study.

Grade Assigned	Interpretation
0	No positive structures
0/+	Occasionally positive structures
+	Few positive structures
+/++	Few to moderate positive structures
++	Moderate positive structures
++/+++	Moderate to numerous positive structures
+++	Numerous positive structures
+++/++++	Numerous to abundant positive structures
++++	Abundant positive structures

**Table 3:** Summary table of results obtained from immunohistochemistry (IHC).

Patient No.	Sex	Age	Ki-67	TUNEL	EGFR	VEGF		IL-1α		IL-10	PGP 9.5
						Epi	Endo	Epi	C.T.		
1	F	7	+	+++	++/+++	++	+++	+++	++	0	0/+
2	F	18	+	++	++	++/+++	+++	+++	++	0	+/++
3	F	28	++	++/+++	+++	+++	++	+++	+++	+	+/++
4	F	32	++	++++	++/+++	+++	++	+++	+++	++	++
5	F	32	+/++	++/+++	++	+++	+++	+++/++++	+++	0/+	+/++
6	F	42	+++	++++	++++	+++	+++	+++	+++	++	0/+
7	F	43	+/++	+++	++	++	++	+++	+++/++++	0	0/+
8	F	45	+/++	+++	+++/++++	+++	+++	+++	++/+++	0	+
9	F	55	++	+++	+++	++	++	+++	+++	+	+
10	F	56	++	+++	+++	+++	+++	+++	+++	+	++
Avg. Vocal Noduli Tissue			++	+++	+++	+++	++/+++	+++	+++	Var 0 to ++	Var 0/+ to ++
Avg. Control Tissue			0/+	+/++	++	0	0/+	0	0/++	+/++	+
Difference (*p* value)			0.0003 **	0.0006 **	0.005 **	0.0003 **	0.0003 **	0.0003 **	0.0003 **	0.050	0.091

** Statistically significant difference between control and vocal noduli tissue (*p* < 0.05; Mann–Whitney *U* test). The interpretation scale for the semi-quantitative scale is provided in Table 2; Abbreviations—Ki-67—marker of proliferation Ki-67; TUNEL—terminal deoxynucleotidyl transferase dUTP nick end labelling; EGFR—epidermal growth factor receptor; VEGF—vascular endothelial growth factor; IL-1α—interleukin-1α; IL-10—interleukin-10; PGP 9.5—protein gene product 9.5; Var—variable expression; Epi—epithelial tissue; Endo—endothelium/blood vessels and C.T.—connective tissue or subepithelial layer.

**Table 4:** Correlation analysis of the vocal noduli tissue factors which were found to have positive and significant (*p* < 0.05) correlations.

Strength of Correlation	Factor 1 **	Factor 2 **	Rho (ρ)	*p* Value
Strong positive correlation (ρ = 0.7–0.9)	Ki-67	TUNEL	0.888	<0.001
	Ki-67	VEGF	0.884	<0.001
	Ki-67	EGFR	0.839	<0.001
	Ki-67	IL-1α (Epithelium)	0.834	<0.001
	Ki-67	IL-1α (Connective tissue)	0.899	<0.001
	TUNEL	VEGF	0.802	<0.001
	TUNEL	IL-1α (Epithelium)	0.786	<0.001
	TUNEL	IL-1α (Connective tissue)	0.820	<0.001
	TUNEL	EGFR	0.753	<0.001
	EGFR	VEGF	0.750	<0.001
	IL-1α (Epithelium)	IL-1α (Connective tissue)	0.868	<0.001
	VEGF	IL-1α (Epithelium)	0.889	<0.001
	VEGF	IL-1α (Connective tissue)	0.745	<0.001
Moderate positive correlation (ρ = 0.5–0.7)	Ki-67	PGP 9.5	0.504	0.032
	EGFR	IL-1α (Epithelium)	0.625	0.005
	EGFR	IL-1α (Connective tissue)	0.611	0.007
	VEGF	PGP 9.5	0.614	0.006
Weak positive correlation (ρ = 0.3–0.5)	IL-1α (Epithelium)	PGP 9.5	0.469	0.049

** Abbreviations: Ki-67—proliferation marker Ki-67, IL-1α-Interleukin-1α, VEGF—vascular endothelial growth factor, PGP 9.5—protein gene product 9.5, EGFR—epidermal growth factor receptor and TUNEL—terminal deoxynucleotidyl transferase dUTP nick end labelling.

## Data Availability

All data analysed in the present study has been presented in a summarized form in the results.
